# Parents’ and peers’ normative influence on adolescents’ smoking: results from a Swiss-Italian sample of middle schools students

**DOI:** 10.1186/s13011-017-0089-2

**Published:** 2017-01-21

**Authors:** Francesca Scalici, Peter J. Schulz

**Affiliations:** Institute of Communication and Health, Università della svizzera italiana, Via Giuseppe Buffi 13, CH-6904 Lugano, Switzerland

**Keywords:** Adolescents, Smoking, Parents’ norms, Peers’ norms, Descriptive norms, Injunctive norms, Conflicting norms

## Abstract

**Background and method:**

Adolescents observe and imitate people to whom they are associated in their social context, and the normative factors sent out by reference groups are crucial determinants of their decision to smoke. The aim of the study is to investigate how adolescents’ smoking changes when they are exposed to factors of pro-smoking normative influence by parents and peers, and how age moderate this relation. A cross sectional survey collected data from 5657 students, aged between 11 and 14, from public and private middle schools in the Italian region of Switzerland (Ticino) on their smoking habits, perceived parents’ and peers’ approval and smoking.

**Results:**

Multinomial logistic regression show that, as adolescents get older, more of the pro-smoking factors come from peers and parents, the higher the risk gets of being a “heavy smoker” has compared against having no experience with smoking. Living in a context with no factor of normative influence toward smoking play a protective role against smoking, and this effect becomes more important than more harmful the smoking behavior in question is. Furthermore, peers’ descriptive norms are more influential for adolescents to become “light” and “heavy smokers”, while smoking being approved by peers is important for adolescents to become accustomed to smoking.

**Conclusions:**

Findings support the different influence of parents’ and peers’ norms on adolescents’ smoking, and highlight the importance of peers’ model behavior as the most important factor influencing smoking during adolescence. Such results have implications for programs that aim to prevent or reduce smoking in early adolescence when friendship choice starts to become crucial.

## Background

Smoking prevalence among adolescents is a factor of major concern in Switzerland as well as in other countries. To study the dynamics that cause adolescents’ tobacco use is an important issue for public health research intending to develop more effective and targeted anti-smoking interventions that aim at the adolescents’ social environments. Adolescents interact with different people and groups, and they are exposed to different behavioral models and opinion coming from different referents in their social environment. Literature is unanimous that adolescents’ exposure to norms coming from their own social environment is the major factor in their taking up smoking [1]. Nevertheless, some gaps are still to be filled. In fact, research so far has treated social norms as single causal agents of adolescents’ tobacco consumption [2–4], but rarely have these norms been researched together, and studied as factors the effects of which might be interacting.

As the social learning theory suggests [5], young people observe and learn by watching the behaviors, and their consequences, of others with whom they live. The adolescents’ decision to embark on a particular behavior depends on the exposure to norms, values and behavioral attitudes of other humans with whom they interact [1, 5–9].

Adolescents’ social environment can be separated into parents and peers (or friends). Behavioral norms are related to adolescents by conversing with both [6–12], and by observing their behaviors [2–4, 12–18]. Normative ideas acquired from conversing are called injunctive, and norms obtained from observing others are called descriptive. Both are crucial in developing the smoking habit [7, 9, 10, 11, 19].

The influence of descriptive norms works through imitation. Adolescents imitate, and are influenced by models who smoke and to whom they are exposed. They smoke more frequently when they are associated with others, family members or peers, who smoke or who have a pro-smoking attitude [9, 10, 11, 19, 20]. Research shows that both types of norms (injunctive and descriptive) and both referents (parents and peers) influence adolescents’ smoking, and play a key role in adolescents’ decision to engage in such behavior [21–27]. There is no agreement which of the referents, parents or peers, exert the stronger influence on adolescents’ smoking behaviors, and neither is there complete agreement about which of the two kinds of norms exerts the stronger impact. Literature agrees smoking by peers is the most important factor predicting tobacco use. Adolescents whose peers smoke are more likely to smoke and to embark on such behavior [28–32]. Similar to peers’ smoking, parents’ smoking is identified as a factor in adolescent smoking [33–35], but this association decreases as adolescents get older [2, 30].

Research provides evidence that supports the influence of peers’ (dis)approval and parents’ (dis)approval on adolescents’ smoking and intention [20–23, 36, 37], but while peer influence increases with adolescents’ age, parent influence decreases [21, 36].

Supported by the literature, we can conclude that both types of norms and both referents have an impact on adolescents’ smoking, and play a key role in adolescents’ decision to engage in such behavior. As evidence suggests, living in an environment where norms from parents and peers are consensual and not in conflict, gives a maximal chance that adolescents will behave according to these norms [4, 11]. If the parents’ and peers’ norms are conflicting, the question arises which side will prevail when it comes to affecting adolescents’ smoking behavior. This study is concerned with two factors of normative influence and their relative impact upon adolescents’ smoking behavior. One factor is the origin of the normative influence, which can come from either parents or peers. The other is the type of norm, which can be either descriptive or injunctive. The influence of the two reference groups and the two types of norms have rarely been researched together. Our study intends to contribute to closing this gap in research in a Central European sample of adolescents. As a new element, we include age as a possible moderator of parents’ and peers’ influence on adolescents’ smoking behaviors, in expectation that parents’ influence decreases and friends’ influence increases as adolescents get older [2, 24, 36, 38–41].

### Hypotheses and research questions

The following hypotheses will be tested:

Adolescents whose parents smoke will themselves smoke more than adolescents whose parents do not smoke (H1).

Adolescents whose peers smoke will themselves smoke more than adolescents whose peers do not smoke (H2).

Adolescents whose peers approve of smoking will smoke more than adolescents whose peers disapprove of it (H3).

Adolescents whose parents approve of smoking will smoke more than adolescents whose parents disapprove of it (H4).

The more factors of pro smoking normative influence an adolescent is exposed to, the more will he or she smoke (H5, cumulative effect).

As said, the study is concerned with the relative influence of origin and types of norms. Lacking a basis for formulating hypotheses, we address that subject as a research question:

Which is the stronger influence on adolescents’ smoking, parent or peer model behavior? (RQ1).

Which is the stronger influence on adolescents’ smoking, peer model behavior or peer approval? (RQ2).

Finally, we argue that with the adolescents’ age the influence of parent norms grows weaker, relatively speaking, while the influence of peer norms gets stronger. In other words, it is hypothesized that age moderates the effect of model behavior as well as of injunctive norms. Formally, we formulate:

As adolescents get older the influence of peer norms increases relative to the influence of parent norms with regard to smoking (H6).

## Method

The study protocol was submitted and approved by the cantonal Department of Education (DECS) because the Università della Svizzera italiana did not have an IRB system in 2011. Data were collected, without asking parental consent, in the Italian region of Switzerland (Ticino) middle schools, after we had received the written cantonal approval.

### Data collection and sample description

The Institute of Communication and Health at the university of Lugano (Università della Svizzera italiana), in collaboration with the Swiss Non-smokers’ Association in Ticino and the DECS, conducted a cross-sectional survey from October 2011 to January 2012. Four rounds of pre-test were carried out in May and June 2011 with 10 students, aged between 11 and 12, from the first and second grades, and with 8 students, aged between 13 and 14, from the third and fourth grades up [36]. Questions related to the socioeconomic status of the respondents’ families were present in the questionnaire at the pre-test phase. Results of pre-test showed that students of the first and the second grade were not able to answer them. The questions were therefore deleted from the final version of the questionnaire.

Students aged between 11 and 14, from all 42 public and private middle schools in Ticino, participated in the survey. Out of a total of 598 classes, 285 were randomly selected, namely 69 for the first grade, 69 for the second grade, 73 for the third grade and 74 for the fourth grade (Table 1). The school director personally informed students about the survey, and teachers detailed the study protocol. Students, after having given their consent to participate, filled in an anonymous paper and pencil questionnaire during the class hours. To ensure anonymity, the completed questionnaire was put in a covered box handed over to the researchers. The questionnaire took 25 min to fill out. A total of 5890 students were members of the selected classes, and 5657 correctly completed questionnaires (response rate 96%) were included in our dataset, which represents the 46.3% of the total middle school students in Ticino (*Total N:* 12210), [36].

**Table 1:** Sample

	N (5657)	%
*Gender*		
Male	2820	49.9
Female	2768	49.0
*Nationality*		
Swiss	4177	73.9
Italian	506	9.0
Others	708	12.5
*Grade*		
First (11 years)	1381	24.4
Second (12 years)	1370	24.3
Third (13 years)	1392	24.6
Fourth (14 years)	1501	26.6

### Measures

The following measures were employed in the analyses reported in this article:

Injunctive norms were measured as *perceived parents’ and peers’ approval,* perceived that is by the respondent student [36].

*Parents’ smoking*: students answered to two items, “Does your father smoke?” and “Does your mother smoke?” Answers were combined in a dummy variable 0 = “neither parent smokes” and 1 = “one or both smoke”.

*Perceived peers’ smoking* was measured with one item, “How many of your friends smoke? Answers were recorded on a 5-point scale from 1 = “all my friends smoke” to 5 = “nobody smokes”. The variable was recoded in a dummy variable 0 = “nobody smokes” and 1 = “my friends smoke (all smoke, the majority smoke, some smoke, few smoke),” in order to be consistent with the other independent variables.

*Respondents’ tobacco consumption* was measured with three items: “have you ever smoked in your life? (Answers were recorded as 0 = “no” and 1 = “yes”); “how frequently do you smoke?” (Answers were recorded on a 4-point scale from 1 = “every day” to 4 = “never); “if you currently smoke, how many cigarettes do you smoke in a week?” (Answers were recorded on a 7-point scale from 1 = “more than one pack” to 7 “none”). The variables were recoded in a single smoking behavior scale with 1 = “never tried, no smoking experience”, 2 = “non-smokers with smoking experience (adolescents who have tried smoking but have not continued)”, 3 = “light smokers (smoke once a week or less and less than 3 cigarettes)”, 4 = “heavy regular smokers (smoke every day or 3 or 4 or more cigarettes in a week).”

*Grade level* was used as representative of the students’ age, ranging from first to fourth grade (11 to 14 years), [36].

### Data analysis

Multinomial Logistic Regression is used as regression analysis to conduct when the dependent variable is nominal with more than two levels. The multinomial regression might be interpret in terms of predictive analysis. It is used to describe data and to explain the relationship between one dependent nominal variable and one or more continuous-level (interval or ratio scale) independent variables.

Sometimes a probit model is used instead of a logit model for multinomial regression. Both models are commonly used as the link function in ordinal regression. However, most multinomial regression models are based on the logit function (like the one implemented in SPSS). The difference between both functions is typically only seen in small samples because probit assumes normal distribution of the probability of the event, when logit assumes the log distribution. At the center of the multinomial regression analysis is the task estimating the k-1 log odds of each category.

As we had a nominal dependent variable with four levels and four independent variables with two levels each, multinomial logistic regression was applied to evaluate the effect of parents or peers approval and parents or peers smoking attitude on adolescents smoking. Hence, after choosing/setting the group of never-smoking adolescents as reference, three models were been estimated comparing respectively “non-smokers with smoking experience” relative to “never-smokers”, “light smokers” relative to “never-smokers” and “heavy smokers” relative to never-smokers. The basic idea is to use, within a general linear model setting, logits as link function. When we use logits we restrict the probability values to (0, 1). Technically this is the log odds (the logarithmic of the odds of y = 1).

## Results

### Overview

Based on *N* = 5649 cases, the independent variables that measure various aspects of normative influence distribute quite differently. More than two in five respondents (42%) declared that at least one of his or her parents smoked. A considerably larger share of adolescents (62%) reported that few or more (some, the majority, all) of their friends smoked. In contrast to this perception, hardly any respondent said that either his or her parents or friends approved of adolescent smoking. In fact, 99% perceived their parents and 97% their friends as disapproving smoking, including taking a neutral position. Only 32 respondents saw their parents and 150 their friends as approving teenage smoking. This lack of variance in the perceptions of approval precludes some analyses we would have liked to run, systematically comparing the influence of injunctive and descriptive norms.

Three quarters of the respondents said they did not smoke and never had, not even tried. Every seventh respondent (14%) had had some experience with smoking but did not classify as smoker presently. Another 4% were classified as light, 5% as heavy smokers.

### Effect of single variables

#### Non-smokers with smoking experience relative to adolescents who never smoke

This chapter compares the probability of having some experience with smoking without developing a habit of it with the probability of having made no experience at all with smoking. Each of the three independent variables—peer disapproval of smoking, non-smoking parents, non-smoking peers—reduces this probability and therefore represents a protective factor against smoking. When a youth has friends who approve of teenage smoking, his or her probability to have tried it (without developing habit of fit) is 1.593 times higher than when friends disapprove (*p* < 0.05). When parents smoke the selfsame probability is 1.794 times (*p* < 0.001) higher than when parents do not smoke, and when peers smoke it is 8.051 times higher than when peers do not smoke.

#### Light smokers relative to adolescents who never smoke

This chapter compares the probability of becoming a light smoker with the probability again of having made no experience at all with smoking. Both descriptive norms—non-smoking parents and non-smoking peers—reduce this probability and therefore represent a protective factor against becoming a light smokers. When a youth’s friends smoke, his or her probability to turn into a light smoker is 23.085 times higher than when friends abstain (*p* < 0.001), and when parents smoke the selfsame probability is 1.743 times (*p* < 0.001) higher than when parents do not smoke. Peer approval of teenage smoking, however, does not play a significant role in predicting who will become a light smoker.

#### Heavy smokers relative to adolescents who never smoke

This chapter compares the probability of becoming a heavy smoker with the probability of having made no experience at all with smoking. Each of the three independent variables—peer disapproval of smoking, non-smoking parents, non-smoking peers—reduces this probability and therefore represents a protective factor against habitual heavy smoking. When a youth has friends who approve of teenage smoking, his or her probability to become a heavy smoker is 4.625 times higher than when friends disapprove (*p* < 0.001). When parents smoke the selfsame probability is 3.154 times (*p* < 0.001) higher than when parents do not smoke, and when peers smoke it is 49.552 times higher than when peers do not smoke (*p* < 0.001).

In general, we can conclude that the protective role of peers’ disapproval and the effect of a smoke-free environment become more and more evident as the “severity” of the adolescent’s smoking behavior increases [from having just tried to developing the habit of light and heavy smoking]. The results support H1, H2, H3, while H4 cannot be tested do to the low number of cases in the sample. The fact that for all three smoking behaviors discussed, the effect is strongest for peers’ descriptive norms, second strongest for parents’ descriptive norms and weakest for peers’ injunctive norms gives a preliminary answer to RQ1 and RQ2.

### Cumulative effects of norms and age interaction

Table 2 presents the results of the multinomial logistic regression for the cumulative effect of norms. The four normative factors that are considered as independent variables in this study are conceptualized as presence or absence of pro-smoking norms. It was hypothesized that adolescents will smoke more the more pro-smoking factors are present in the environment they live in. To test this assumption the number of pro-smoking factors in a person’s environment was added up, ranging theoretically from 0 to 4, but not a single respondent indicated he or she was living in an environment with all four pro-smoking factors present.

**Table 2:** Significant cumulative effects of parents’ and peers’ norms and age interaction

			Non-smokers with smoking experience				Light smokers				Heavy smokers			
			*B*	*Std. Error*	*p*	*Odds*	*B*	*Std. Error*	*p*	*Odds*	*B*	*Std. Error*	*p*	*Odds*
*Pro-smoking factors*	vs	Parents and peers neither smoke nor approve												
Peers smoke (1 factor)			1.566	.197	.000	4.790	2.585	.521	.000	13.257	2.174	.597	.000	8.798
Parents smoke (1 factor)			.821	.245	.001	2.273	.302	.766	.693	1.351	-.430	1.157	.710	.651
Peers smoke and approve (2 factors)			1.995	.390	.000	7.352	2.901	.789	.000	18.200	4.231	.674	.000	68.811
Parents and peers smoke (2 factors)			2.185	.196	.000	8.889	3190	.519	.000	24.286	3.506	.590	.000	33.307
Parents smoke, peers smoke and approve (3 factors)			2.676	.400	.000	14.532	3.693	.746	.000	40.154	4.778	.689	.000	118.868
Grade			.577	.048	.000	1.781	.352	.081	.000	1.442	.827	.088	.000	2.286
*Age interaction*														
Peers and parents smoke* Grade			.013	.127	.917	1.013	.188	.220	.394	1.206	.805	.229	.000	2.236
Peers smoke and approve* Grade			-.187	.363	.607	.830	.096	.592	.871	1.101	1.250	.389	.001	3.490
Parents smoke, peers smoke and approve* Grade			.576	.363	.113	1779	-.862	.862	.317	.422	1.407	.453	.002	4.083

Presents the results of the multinomial logistic regression for the cumulative effect of norms

* = indicate the product term for the interaction

The lowest risk to show any type of smoking behaviors against having no experience was reached by the group of adolescents who reported that neither their parents nor their peers smoked and neither approved of it, or in other words, by the group with no factor of normative influence directing them towards smoking (non-smoker with smoking experience: OR = 14.532, p < 0.001; light smoker: OR = 40.154, *p* < 0.001; heavy smoker: OR = 118.868, *p* < 0.001), (Table 2).

The two groups in between reach the higher risk to be in one category of smokers the more pro factors are present.

With one normative factor present, the probability of either of the three smoking behaviors against having no experience with smoking become higher as smoking behavior becomes more severe, but only for adolescents having smoking peers (non-smoker with smoking experience: OR = 4.790, *p* < 0.001; light smoker: OR = 13.257, *p* < 0.001; heavy smoker: OR = 8.798, *p* < 0.001). For adolescents having smoking parents the change in the odds is only significant in the case of “non-smoker with smoking experience” compared to “never smokers” (OR = 2.273, *p* < 0.01), while it is not significant for “light smokers” and “heavy smokers”, (Table 2).

With two factors present, the probability of either of the three smoking behaviors against having no experience with smoking become higher as smoking behavior becomes more severe. This is true for adolescents having approving and smoking peers (OR = 7.352, *p* < 0.001; OR = 18.200, *p* < .001; OR = 68.111, *p* < 0.001) and for those having smoking parents and peers (OR = 8.889, *p* < .001; OR = 24.286, *p* < .001; OR = 33.307 *p* < 0.01) relative to those that are in the group with no factor of normative influence, (Table 2).

In line with H5, we can conclude that the protective role of living in a context with no factor of normative influence toward smoking becomes more important than more harmful the smoking behavior in question is.

Moreover, adolescents tend to smoke more intensely as they get older. Increasing age significantly predicts the three smoking behaviors against having never smoked, and the effect increases, as the smoking behavior in question becomes more harmful. Nevertheless, the interaction model suggests that increasing age does not change the cumulative effect of normative factors for “non-smokers with smoking experience” and for “light smokers” relative to “never-smokers, while is a contributing factor for becoming a “heavy smoker” (Table 2). As adolescents get older, the risk of being a “heavy smoker”, given one unit of age increase, is by far most pronounced in a normative environment where both parents and peers smoke and peers approve (OR = 4.083, *p* < 0.01), where both referents smoke (OR = 2.236, *p* < 0.001), and where peers approve and smoke (OR = 3.490, *p* < 0.01), (Table 2). The results in part confirms H6. As adolescents get older, more of the pro-smoking factors come from peers and parents have, and the higher the risk gets of being a “heavy smoker” has compared against having no experience with smoking.

### Comparison of effects in conflicting situations

This analysis first compares adolescent smoking behavior in two situations in which parents and peers showed opposing model behavior with regard to smoking. Realistically assuming that the intensity of adolescent smoking behavior in situations with conflicting normative factors lies between the consonant situations of neither group smoking and both groups doing it, the question is simply, which of the two conflicting situations produces more smoking in adolescents. If adolescents who live in an environment that shows smoking peers and nonsmoking parents smoke more than their counterparts who know smoking parents and nonsmoking peers, peer model behavior is considered the more influential factor.

A first multinomial logistic regression with a model interaction was run to compare conflictual situations of the two referent groups within one type of norm (Table 3). The highest risk to be a “non-smoker with smoking experience”, a “light smoker” or a “heavy smoker” was reached by the group of adolescents who reported their parents and peers smoke (Table 3). In case of conflictual situations, where parents smoke and peers do not smoke, the change in the odds is significant only for “non-smokers with smoking experience”, while it is not significant for the other two smoking behaviors (Table 3). This means that the influence of parents’ descriptive norms disappears as the smoking behavior in question becomes more harmful. In the opposite situation, when the conflict consists in having smoking peers and non-smoking parents, the probability of any type of smoking behaviors against having no experience become higher as smoking behavior becomes more severe (non-smoker with smoking experience: OR = 8.835, *p* < 0.001; light smoker: OR = 20.025, *p* < 0.001; heavy smoker: OR = 22.893, *p* < 0.001), (Table 3).

**Table 3:** Comparison of effects in situations of conflicting norms

			Non-smokers with smoking experience				Light smokers				Heavy smokers			
			*B*	*Std. Error*	*p*	*Odds*	*B*	*Std. Error*	*p*	*Odds*	*B*	*Std. Error*	*p*	*Odds*
*Within norms conflict between referents*														
Peers smoke, parents do not smoke	vs	Parents and peers do not smoke	2.179	.187	.000	8.835	2.997	.513	.000	20.025	3.131	.590	.000	22.893
Peers do not smoke, parents smoke		Parents and peers do not smoke	.743	.240	.002	2.103	.264	.765	.731	1.302	-.555	1.156	.631	.574
Peers and parents smoke		Parents and peers do not smoke	2.745	.187	.000	15.570	3.562	.512	.000	35.228	4.307	.585	.000	74.208
Peers approve, parents do not approve		Parents and peers do not approve	.465	.236	.049	1.592	.390	.409	.340	1.478	1.534	.255	.000	4.638
*Between norms conflict within one referent*														
Peers approve and smoke		Peers do not approve and do not smoke	2.504	.269	.000	12.236	3.509	.553	.000	33.404	5.387	.557	.000	218.530
Peers do not approve and smoke			2.105	.129	.000	8.210	3.128	.387	.000	22.837	3.870	.506	.000	47.945
Peers approve and do not smoke			1.184	.782	.059	4.392	-	-	-	-	-	-	-	-

Show results of a first multinomial logistic regression with a model interaction that compare the conflictual situations of the two referent groups within one type of norm, and of a second multinomial logistic regression that compare the influence of two types of norms within one type of referent

A similar analysis compares the injunctive norms of both referent groups that is parent vs. peer approval of adolescent smoking. The probability of being a “non-smoker with smoking experience” rather than a “never smoker” is 1.591(*p* < .05) times higher for adolescents having peers that approve and parents that do not approve relative to those where both disapprove smoking. This effect is not significant for “light smokers” relative to “never smokers”, while it became highly significant for “heavy smokers” relative to “never smokers” (OR = 4.638, *p* < 0.001).

These calculations support the assumption that peer model behavior of smoking is clearly more influential than smoking parents.

A similar logic can be applied to compare the influence of two types of norms within one type of referent. It can, however, be done only for peers as all 32 cases in which adolescents perceived their parents as approving of their smoking had missing cases for parents’ smoking behavior. This precludes a comparative analysis of parent injunctive vs. descriptive norms.

Again, the higher probability to be “non-smokers with smoking experience”, “light-smokers” or “heavy-smokers” rather than “never smokers” is reached by the group where peers approve smoking and smoke, and this probability becomes higher as the smoking behavior in question becomes more serious (Table 3). In case of conflict between peers’ norms (i.e. peers do not approve and smoke) the probability of either of the three smoking behaviors against having no experience with smoking become higher as smoking behavior becomes more severe (non-smoker with smoking experience: OR = 8.210, *p* < 0.001; light smoker: OR = 22.837, *p* < 0.001; heavy smoker: OR = 47.945,*p* < 0.001). In the opposite situation (i.e. peers approve and do not smoke), the change in the odds is significant only for non-smoker with smoking experience (OR = 8.210, *p* = .05), (Table 3).

Similar analysis for injunctive norms would be desirable but could be not computed due to the mentioned missing data.

In summary, the comparisons of the intensity of adolescent smoking in situations of conflicting normative influence show that peer model behavior is more influential than parent model behavior and also more influential than peer injunctive norms, with somewhat weaker evidence in the latter case.

The matter of interest, however, is the comparison between the environments where only peers’ respectively only parents’ smoke. It is obvious that the model behavior of peers is more influential in driving adolescents towards tobacco use than bad examples their parents give.

Furthermore, results shows that peers’ behaviors (i.e. descriptive norms) are more influential for adolescents to become “light” and “heavy smokers”, while smoking being approved by peers is important also for adolescents to become accustomed to smoking.

## Discussion and conclusions

Summarizing our findings, this research shows that each of four normative factors increased the inclination in adolescents aged 11 to 14 to smoke. The four factors are parents’ and peers’ model behavior, also referred to as descriptive norms, and their approval respectively disapproval of adolescent smoking, also referred to as injunctive norms. Smoking parents and peers as well as the expression of approval by these referents drives adolescents towards (increased or newly begun) tobacco consumption. The influence of these factors is cumulative; that is each of them has something to contribute to adolescent smoking. The issue of which is the most influential (and that means detrimental) factor can be answered only on incomplete evidence. It can be fairly certainly said that peer model behavior contributes to adolescents smoking more than parent model behavior and more than peer injunctive norms. That is to say, what one’s pals do and show when it comes to smoking impresses more than what they think and say, and it is also more consequential than what parents do. Moreover, being already superior to parent influence, peer influence further gains ground for the regular smokers as children get older.

Our paper has the advantage of including simultaneously multiple causal agents that contribute to create a pro or anti-smoking environment. In contrast to the existing literature, we did not look at the effect on adolescents’ smoking of a single social norm, but of relevant cases of different, partly conflicting norms. This allowed us to identify the stronger force when adolescents are exposed to conflictual or not consensual norms from their reference group. In that perspective, the analysis shows a pervasive influence of peer model behavior on young adolescents’ smoking habits. This influence seems to increase, as the seriousness of smoking behavior increases, with age. The other two normative factors, peers’ attitudes to smoking and parent model behavior do not affect adolescents’ smoking in a comparable degree.

The analysis helps to enrich the debate regarding social norms and smoking, and to strengthen some points already established in the literature. In an environment where parents and peers are the main points of reference of adolescents, their norms and behaviors are both contributing factors of adolescents’ smoking, with peers’ normative model being a major determinant of adolescents’ decision to smoke [9, 18, 21–29].

In line with research that states smoking is more likely to occur when norms from referents are uniformly pro-smoking [10], in our sample, adolescents who live in a consensual pro-smoking environment where peers’ and parents’ opinion and behavior are all pro-smoking are more likely to smoke than those who live in a non-smoking normative environment. The simultaneous study of conflicting social norms suggests that, when the different factors of normative influence work and interact, the strength of the influence of parents’ and peers’ behaviors and opinions is different. In the debate on the role of parents and peers’ social norms on adolescents’ smoking, our results clearly support and reinforce the part of the literature that affirms adolescents behave based on what they see in their social environment, and they imitate, primarily, their peers [24, 28, 29, 40].

Nevertheless, our findings should be read in light of some limitations. First, the most important weakness of the paper is that the measures of parents’ and peers’ smoking and approval are as perceived by adolescents. Smoking among others is often overestimated by adolescents and depends on adolescents’ own smoking [40–42]. Moreover, the communication of disapproval of a behavior, from the parent to their child, is part of a communication process that, during adolescence, can be complex and influenced by many factors. Moreover, it is just the complexity of this communication process that often gives rise to a gap between parents’ message and adolescents’ perception of it [43, 44]. Second, due to the cross-sectional methodology, we cannot draw conclusions about the causality of relationships. Third, the lack of information about the respondents’ socio-economic status did not allow us to include in the tested models these variables. Socio-economic factors (SES), namely education level, income and/or occupational status [45], are strong predictors of smoking among adolescents. Past literature agrees that there is an inverse relationship between SES and adolescents’ smoking; the higher the household income is, and the higher the parental education level is, the lower the possibility that children smoke [46–49]. The analysis of the effect on adolescents’ smoking of the interaction among SES and social norms should be investigated in future studies. Finally, the lack of data concerning the measure of parents’ approval does not allow us to draw firm conclusions concerning the role of parents’ opinion.

Despite the limitations, the large sample size, the simultaneous inclusion of peers’ and parents’ descriptive and injunctive norms, and the examination of a Central European sample of adolescents, represent the major strengths of this paper, and our findings can have some implications for public health policies. Together with other measures and policies implemented by public and private actors active in the tobacco consumption prevention, our conclusions support the implementation of smoking prevention programs, starting from early adolescence, that intervene in the social environment adolescents live in. Adolescents smoke because they imitate other people they meet in private or public places they usually frequent. In that sense, we suggest tobacco prevention programs targeting adolescents and parents, addressed in schools and in public places that first help adolescents to avoid smoking initiation by making them attentive to the role that friends play in their choice to smoke. Second, programs that aim to improve the role of parents during adolescence making them aware that their intervention, communication and monitoring can be crucial in reducing the risk for their children to become smokers in early adolescence [50], and by providing them information about the importance of monitoring who their children are friends with. The cumulative nature of the effects of the normative factors suggests that no point of attack is completely futile when it comes to fighting juvenile tobacco consumption.
